# Alterations in glucose metabolism in *Vibrio cholerae* serogroup O1 El Tor biotype strains

**DOI:** 10.1038/s41598-019-57093-4

**Published:** 2020-01-15

**Authors:** Donghyun Lee, Eun Jin Kim, Yeongjun Baek, Jiwon Lee, Youngbae Yoon, G. B. Nair, Sang Sun Yoon, Dong Wook Kim

**Affiliations:** 1 Department of Pharmacy, College of Pharmacy, Hanyang University, Ansan, 15588 Republic of Korea; 2 Institute of Pharmacological Research, Hanyang University, Ansan, 15588 Republic of Korea; 3 Microbiome Laboratory, Rajiv Gandhi Centre for Biotechnology, Kerala, 695014 India; 4 Department of Microbiology and Immunology, Yonsei University College of Medicine, Seoul, 03722 Korea; 5 Brain Korea 21 PLUS Project for Medical Sciences, Yonsei University College of Medicine, Seoul, 03722 Korea; 6 Institute for Immunology and Immunological Diseases, Yonsei University College of Medicine, Seoul, 03722 Korea

**Keywords:** Bacterial infection, Diarrhoea

## Abstract

The 2 biotypes of *Vibrio cholerae* O1 serogroup strains—classical and El Tor—use glucose in distinct ways. Classical biotype strains perform organic acid-producing fermentation and eventually lose viability due to the self-induced creation of an acidic environment, whereas El Tor biotype strains use an alternative neutral fermentation pathway, which confers them with survival advantages. However, we report that the neutral fermentation pathway has only been recruited in prototype Wave 1 El Tor biotype strains, which have not been isolated since the mid-1990s. Current Wave 2 and Wave 3 atypical El Tor strains contain a single-base deletion in a gene that directs bacteria toward neutral fermentation, resulting in the loss of neutral fermentation and an appearance that is similar to classical biotype strains. Moreover, when sufficient glucose was supplied, Wave 1 El Tor strains maintained their use of acid-producing fermentation, in parallel with neutral fermentation, and thus lost viability in the late stationary phase. The global replacement of Wave 1 El Tor strains by Wave 2 and 3 atypical El Tor strains implies that the acidic fermentation pathway may not be disadvantageous to *V. cholerae*. The characteristics that we have reported might improve oral rehydration in the treatment of cholera.

## Introduction

The O1 serogroup *Vibrio cholerae* has been classified into 2 biotypes: classical and El Tor^1^. Both biotypes of bacteria cause the life-threatening diarrheal disease cholera by producing cholera toxin (CT)^2^. Classical and El Tor biotype strains synthesize biotype-specific CT, which differs by 2 amino acids of the 124 that constitute the CT binding subunit (CTB)^3^.

Of the 7 cholera pandemics that have been acknowledged since the early 19^th^ century, the first 6 were caused by classical biotype strains, whereas El Tor biotype strains are responsible for the current seventh pandemic, which began in 1961^4^. Since the emergence of El Tor biotype strains, classical biotype strains have not been detected among clinical isolates. This change in *V. cholerae* population has remained a contradiction, of which a difference in glucose metabolism between the 2 biotype strains has been widely accepted to be a cause^5^. El Tor biotype strains use glucose by neutral fermentation, whereas classical biotype strains perform acidic fermentation of glucose, which is considered an advantage for the former with regard to survival in a host and perhaps in nature^5,6^.

The molecular mechanism of this difference in glucose metabolism has been deduced by characterizing the function of 3 genes—VC1589 (*alsD*, acetolactate decarboxylase), VC1590 (*alsS*, acetolactate synthase), and VC1591 (*alsO*, oxidoreductase)—that regulte the neutral fermentation that produces 2,3-butanediol in El Tor biotype strains. The classical biotype strains are unable to generate neutral fermentation products, perhaps due to 2 point mutations in the regulatory gene VC1588 (*alsR*, a transcriptional regulator of the alpha-acetolactate operon) and 1 point mutation in VC1590^5,7^.

There has been another population change among El Tor biotype strains since they first emerged in 1961^3^. From 1961 to the early 1990s, prototype El Tor biotype strains, or Wave 1 El Tor strains, which produced the El Tor biotype-specific CTB (*ctxB3*), had been prevalent. However, atypical El Tor strains, or Wave 2 and Wave 3 strains, that synthesized classical type CTB (*ctxB1*) or Haitian type CTB (*ctxB7*) have predominated since the mid-1990s. Prototype El Tor biotype strains have not been isolated since the appearance of atypical El Tor strains^8^. In addition, many atypical El Tor strains have been shown to be negative or weakly positive in the Voges-Proskauer test, which implies that they lack neutral fermentation during glucose metabolism^7,9^.

In this study, we examined the differences in glucose metabolism in prototype and atypical El Tor strains. Although prototype El Tor biotype strains have been recognized to perform neutral fermentation in the presence of glucose in media, the effects of neutral fermentation last for less than 20 hours, and the bacteria eventually shift to acidic fermentation mode and lose viability. Depending on the availability of glucose, prototype El Tor biotype strains perform neutral and acidic fermentation simultaneously, resulting in the accumulation of acidic products and the loss of viability.

We found that the VC1589 gene is nonfunctional in atypical El Tor strains: thus, they only perform acidic fermentation in the presence of glucose. A drop in pH and a loss of viability were observed with atypical El Tor strains in the early culture phase, as in classical biotype strains. The cause of this alteration in phenotype was confirmed by replacing the nonfunctional VC1589 in atypical El Tor strains with the functional VC1589 in prototype El Tor strains, and vice versa. When the nonfunctional VC1589 in atypical El Tor strains was replaced by the functional VC1589 of prototype El Tor strains, the bacteria performed neutral fermentation in the early culture phase and then underwent acidic fermentation, decreasing the pH and viability in the late culture phase. The opposite changes in phenotype were observed in prototype El Tor strains when their functional VC1589 was replaced by the nonfunctional VC1589 of atypical El Tor strains.

Our results imply that the acidic fermentation mode is not entirely disadvantageous to *V. cholerae*. The effects of alterations in glucose metabolism should be detailed to determine the impact of glucose metabolism on the virulence of bacteria. The loss of viability in the presence of glucose by acidic fermentation might be a characteristic that can be exploited in the treatment of cholera by oral rehydration.

## Results

### Bacterial growth in LB media

**Figure 1:**
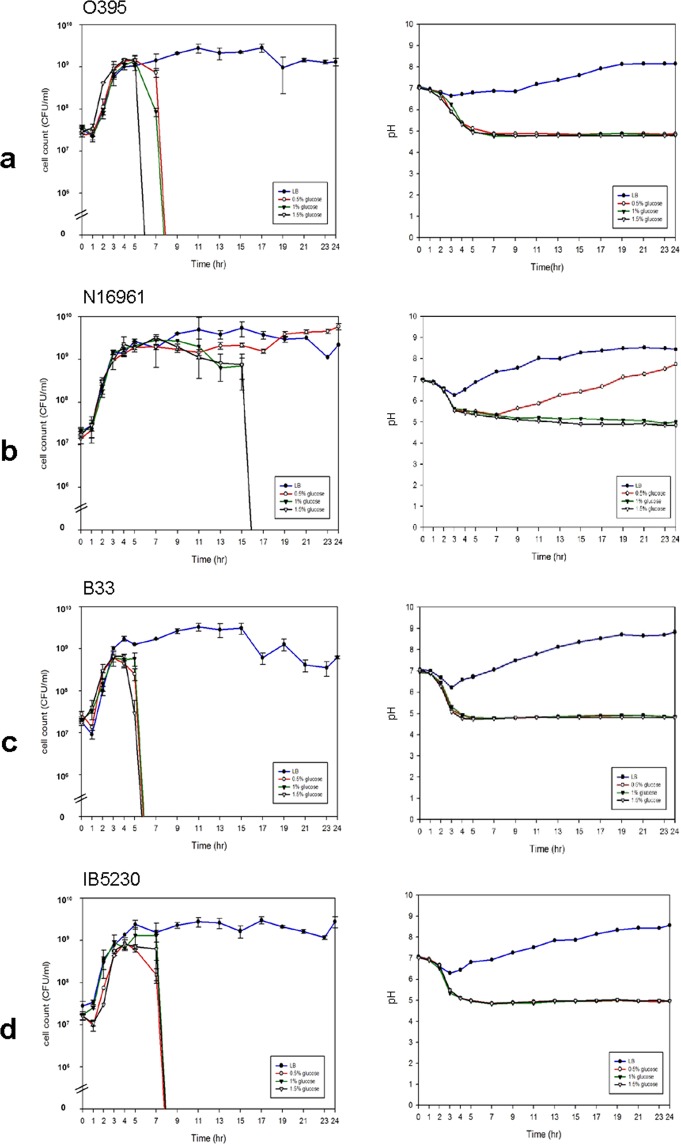
Growth curve (left) and pH changes in cultures (right) of *V. cholerae* strains in glucose-Supplemented Media. (**a**) Classical biotype strain O395, (**b**) Wave 1 prototype El Tor strain N16961, (**c**) Wave 2 atypical El Tor strain B33, and (**d**) Wave 3 atypical El Tor strain IB5230. Glucose was added to LB media at 0% (filled circle), 0.5% (circle), 1.0% (inverted filled triangle), and 1.5% (inverted triangle). Three independent experiments were performed. Values of mean ± standard deviation (viable cell count) and a representative result (pH) are shown.

The growth and survival of a classical biotype strain, O395, and various El Tor biotype strains were monitored in regular LB media and LB media that were supplemented with 0.5%, 1.0%, and 1.5% glucose. In LB media, *V. cholerae* strains showed a typical growth pattern (Fig. 1). Under our culture conditions, they reached the stationary phase in 4–5 hrs after being inoculated in fresh media. The change in pH in the culture media was also recorded. The pH of the media was initially neutral (pH 7.0) and decreased slightly (classical strain approximately pH 6.6 and El Tor strains pH 6.0~6.5) in the first 3–4 hrs and then rose to pH 8.0. This pattern was similar between classical and El Tor biotype strains in LB media.

### Classical biotype strains lose viability in the early stationary phase in glucose-supplemented media

Classical biotype strains lose their viability in the presence of glucose in media^5^. To confirm the growth pattern of classical biotype strains in the presence of glucose, the survival of bacteria and changes in the pH of the culture media were monitored. The classical biotype strain O395 reached the stationary phase in 4–5 hours in LB media that was supplemented with glucose, similar to when they were cultured in regular LB media. As shown earlier, the classical biotype strain started to lose viability when they reached the stationary phase. After 7 hours, the bacteria began to lose viability sharply, and no viable cells remained by Hour 9 (Fig. 1a). The pH of the media declined to pH 4.9 to 4.6 when 0.5%, 1.0%, and 1.5% glucose were added to the culture. These results confirmed the vulnerability of classical biotype strains in acidic conditions that are generated by the acidic fermentation of glucose in media.

### Prototype El Tor biotype strains lose viability in the late growth phase in the presence of high glucose concentrations

Two prototype El Tor strains, N16961 and T19479 (Fig. 1b and Supplementary Fig. S1a), have been tested for viability in the presence of glucose in media^10,11^. Prototype El Tor strains, or Wave 1 El Tor strains, survive in media that is supplemented with glucose, whereas classical biotype strains lose viability under the same conditions due to acidic fermentation. Wave 1 strains reached the stationary phase within 5 hrs in LB media, similar to classical biotype strains (Fig. 1b). The pH of the culture media fell slightly to pH 6.0–6.2 within the first 3 hours compared with classical biotype strains, which decrease to approximately pH 6.5.

Wave 1 El Tor biotype strains behaved differently according to the available glucose in the media. In the presence of 0.5% glucose, the growth pattern of Wave 1 El Tor biotype strains differed from that of classical biotype strains, reaching the stationary phase in 5 hours and maintaining viability for 24 hrs. The pH declined further down to pH 5.5 in the first 4 hrs and then recovered to pH 7.5. When glucose was added to the media at higher concentrations, the Wave 1 El Tor biotype strains reached the stationary phase within 5 hrs and maintained viability for up to 16 hrs. However, they began to lose viability sharply from 16 hrs. The medium became further acidic (pH 4.6) at glucose concentrations above 1.0% (Fig. 1b and Supplementary Fig. S1a).

Based on these results, the growth patterns of Wave 1 El Tor strains differ, depending on the amount of glucose in the medium. When the glucose concentration is higher than 1%, the pH of the culture decreases lower than pH 5, and bacteria lose viability after 17 hrs. Wave 1 El Tor biotype strains have been shown to survive in the presence of 1% glucose in the media, but their survival was monitored only up to the early stationary phase. In this study, Wave 1 El Tor biotype strains eventually lost viability (perhaps due to acidic fermentation) in media that contained more than 1% glucose.

### Loss of viability of Wave 2 and 3 El Tor biotype strains in the presence of glucose

Three Wave 2 El Tor strains—B33, MJ1236, and MG116025^12–14^, and two Wave 3 El Tor strains—IB4122 and IB5230—were acquired to monitor their viability in the presence of glucose in media^15,16^. IB5230 is the 2010 Haitian cholera outbreak strain^17^. All strains had similar growth patterns in LB media: they reached the stationary phase in 4–5 hrs, and the pH fell from 7 to 6 within 3 hrs and then recovered to pH 8 (Fig. 1c,d and Supplementary Fig. S1b–d). However, the change in viability was more similar to that observed in classical biotype strains than in the Wave 1 El Tor strains when glucose was added to the media. The Wave 2 El Tor strains lost their viability in 5–7 hrs in the presence of 0.5% to 1.5% glucose, and this loss of viability was slightly delayed in Wave 3 El Tor strains (7–9 hrs in IB5230 and 9–11 hrs in IB4122). The pH of the culture media fell below 5 (Fig. 1c,d, Supplementary Fig. S1).

### A single-base deletion in VC1589 alters glucose metabolism in Wave 2 and 3 El Tor strains

Because Wave 2 and 3 strains had similar growth patterns as classical biotype strains in the presence of glucose in the media, we expected that the neutral fermentation pathway in Wave 2 and 3 strains differed from that in Wave 1 El Tor strains. VC1589–1591 is critical in the switch in the metabolism of El Tor biotype strains^5^. We sequenced VC1589 and VC1590 in Wave 2 and 3 strains and identified a single-base deletion in VC1589. VC1589 in Wave 1 El Tor strains consists of 785 bp, with 7 thymines from nucleotides 309 to 315; however, VC1589 in Wave 2 and 3 strains contains 6 thymines at this location. VC1589 in Wave 1 strains encodes a 261-amino-acid product (α-acetolactate decarboxylase), and VC1589 in Wave 2 and 3 strains encodes a 116-amino acid nonfunctional, truncated product. Hereafter, the functional VC1589 allele will be designated VC1589-T7, and the nonfunctional VC1589 in Wave 2 and 3 strains will be referred to as VC1589-T6.

**Figure 2:**
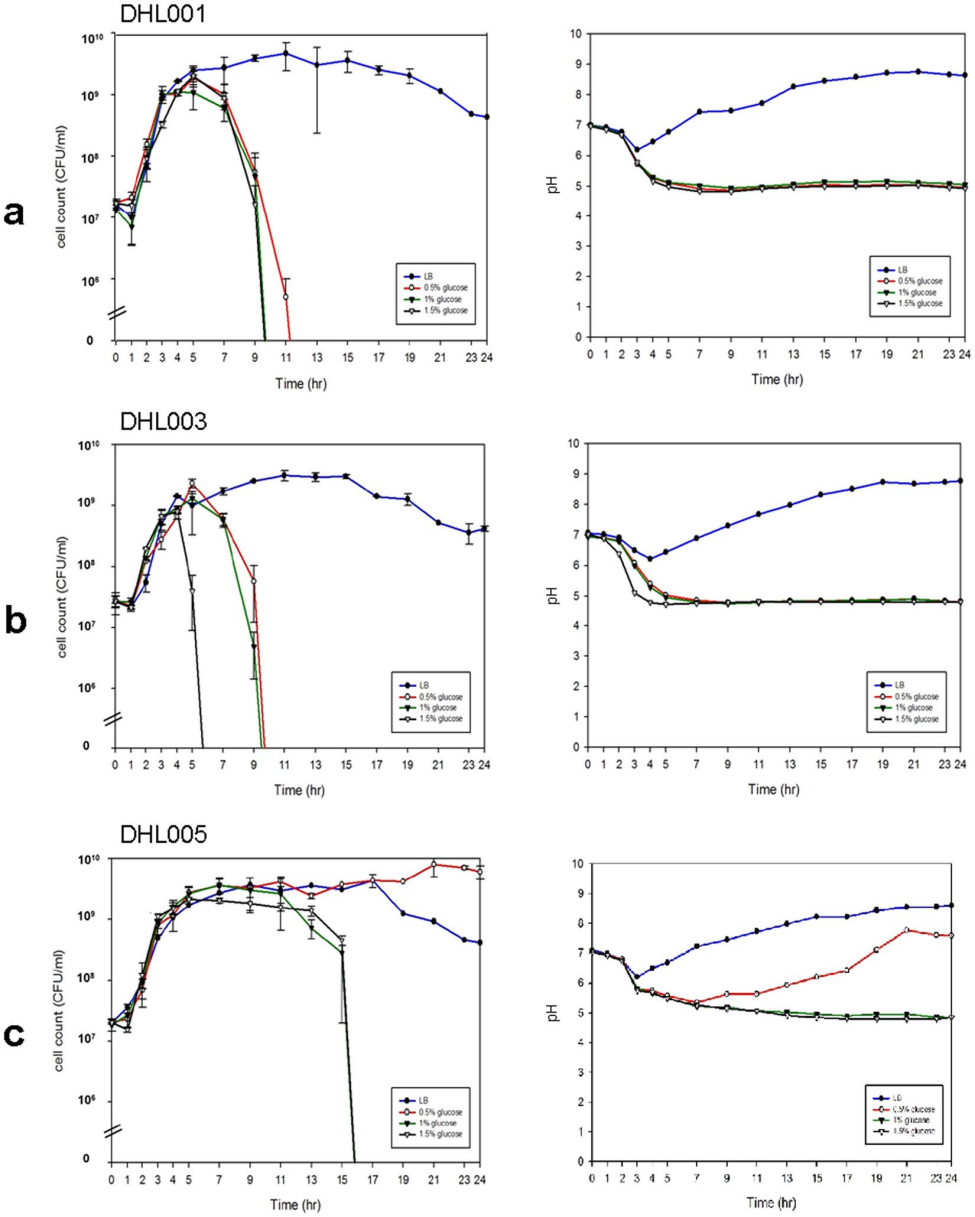
Alterations in glucose metabolism in El Tor biotype strains by a single-base deletion in VC1589: Wave 1 and 2 strains. Growth curve (left) and pH changes in cultures (right) of El Tor biotype strains in glucose-Supplemented Media. (**a**) Derivative of Wave 1 El Tor strain N16961 containing nonfunctional VC1589 (strain DHL001), (**b**) derivative of a Wave 2 atypical El Tor strain B33 containing functional VC1589 (strain DHL003), and (**c**) derivative of Wave 2 El Tor strain MG116025 containing functional VC1589 (strain DHL005). Glucose was added to LB media at 0% (filled circle), 0.5% (circle), 1.0% (inverted filled triangle), and 1.5% (inverted triangle). Three independent experiments were performed. Values of mean ± standard deviation (viable cell count) and a representative result (pH) are shown.

To confirm that the alteration in the use of glucose by Wave 2 and 3 El Tor strains was due to this single-base deletion in VC1589, we constructed derivatives of Wave 1 strains, in which the functional VC1589-T7 was replaced by the nonfunctional VC1589-T6 of Wave 2 and 3 strains by allele exchange method. Similarly, derivatives of Wave 2 and 3 strains that contained functional V1589-T7 were also constructed. Derivatives of Wave 1 strains that contained VC1589-T6 (DHL001 and DHL002) had similar characteristics as Wave 2 and 3 strains. These strains lost their viability earlier (at 7–9 hrs), and the pH of the culture media decreased to 5 in the presence of 0.5 to 1.5% glucose (Fig. 2a and Supplementary Fig. S2a). These results demonstrate that the alteration in glucose metabolism in Wave 2 and 3 strains is caused primarily by a single-base deletion in VC1589.

### Recovery of neutral fermentation in Wave 2 and 3 strains by functional VC1589-T7

We examined the possibility of reverting the acidic fermentation phenotype of Wave 2 and 3 strains to the neutral fermentation phenotype by replacing nonfunctional VC1589-T6 with functional VC1589-T7. The phenotype restoration in the Wave 2 strains is complicated and strain-dependent.

The glucose utilization phenotype of DHL003 (B33-VC1589-T7) and DHL004 (MJ1236-VC1589-T7) remained the same as the parental strains that contained VC1589-T6 (Fig. 2b and Supplementary Fig. S2b). They still lost viability, even in the presence of 0.5% glucose in the media, and there was no delay in viability loss in media that was supplemented with 1.0% and 1.5% glucose, perhaps due to the greater number of alterations in carbohydrate fermentation that facilitated the production of acidic products in these Wave 2 strains. DHL005 (MG116025-VC1589-T7) behaved like Wave 1 strains—this strain can survive in LB media that is supplemented with 0.5% glucose, and the loss of viability in the media that contained 1.0% and 1.5% glucose was delayed to 17 hr (Fig. 2c).

**Figure 3:**
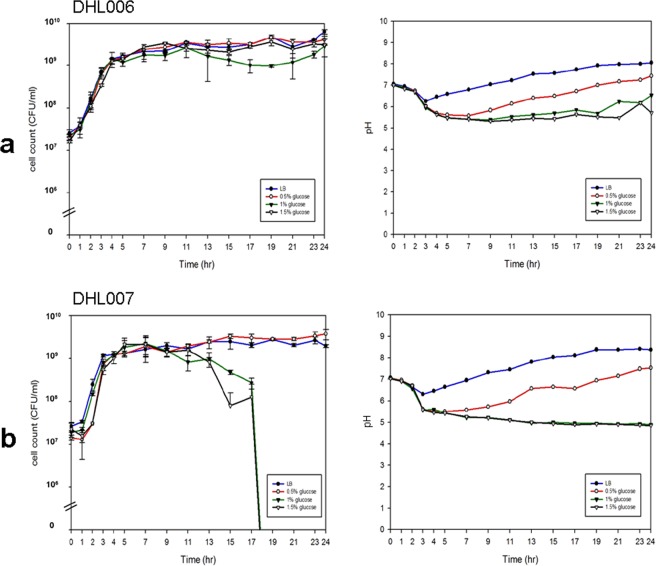
Alterations in glucose metabolism in El Tor biotype strains by a single-base deletion in VC1589: Wave 3 strains. Growth curve (left) and pH changes in cultures (right) of Wave 3 El Tor strains in glucose-Supplemented Media. (**a**) Derivative of Wave 3 El Tor strain IB4122 containing functional VC1589 (strain DHL006) (**b**) derivative of Wave 3 El Tor strain IB5230 containing functional VC1589 (strain DHL007). Glucose was added to LB media at 0% (filled circle), 0.5% (circle), 1.0% (inverted filled triangle), and 1.5% (inverted triangle). Three independent experiments were performed. Values of mean ± standard deviation (viable cell count) and a representative result (pH) are shown.

The recovery of neutral fermentation in Wave 3 strains also differs between strains. The IB4122 Vietnam cholera outbreak strain survived even in high glucose concentrations for up to 24 hrs when the nonfunctional VC1589-T6 was replaced by functional VC1589-T7 (DHL006). It appears that neutral fermentation dominates over the acidic fermentation pathway in this strain (Fig. 3a).

When the IB5230 Haitian strain was manipulated to harbor functional VC1589-T7 (DHL007) it developed a similar phenotype as Wave 1 strains. This strain maintained viability for up to 24 hrs in media that was supplemented with 0.5% glucose. In media with 1.0% and 1.5% glucose, the loss of viability was delayed to 17 hr (Fig. 3b).

Overall, the results confirm that Wave 2 and 3 strains are defective in the neutral fermentation of glucose, due to a single-base deletion in VC1589.

### The rate of pH drop is linked to the rate of loss of viability in *V. cholerae* strains

Based on the results above, the viability loss patterns of *V. cholerae* strains in the presence of glucose can be classified into 2 groups, with several exceptions (Supplementary Fig. S3). *V. cholerae* strains lose their viability within 9 hours or faster, even at a low concentration of glucose (0.5%). Strains with nonfunctional VC1589 (Wave 2 and 3 strains and Wave 1 strains in which the functional VC1589 has been replaced by nonfunctional VC1589) showed this pattern, implying that the rapid decline in pH and viability are related to the disruption of neutral fermentation.

Further, a group of strains can survive at 0.5% glucose but lose viability in the late stationary phase at high glucose concentrations (above 1.0%). Wave 1 strains and certain Wave 2 and 3 strains that harbor functional VC1589, allowing them to perform neutral fermentation, developed this pattern. However, these strains accumulated acidic products and lost viability in the late stationary phase when glucose was abundant.

**Figure 4:**
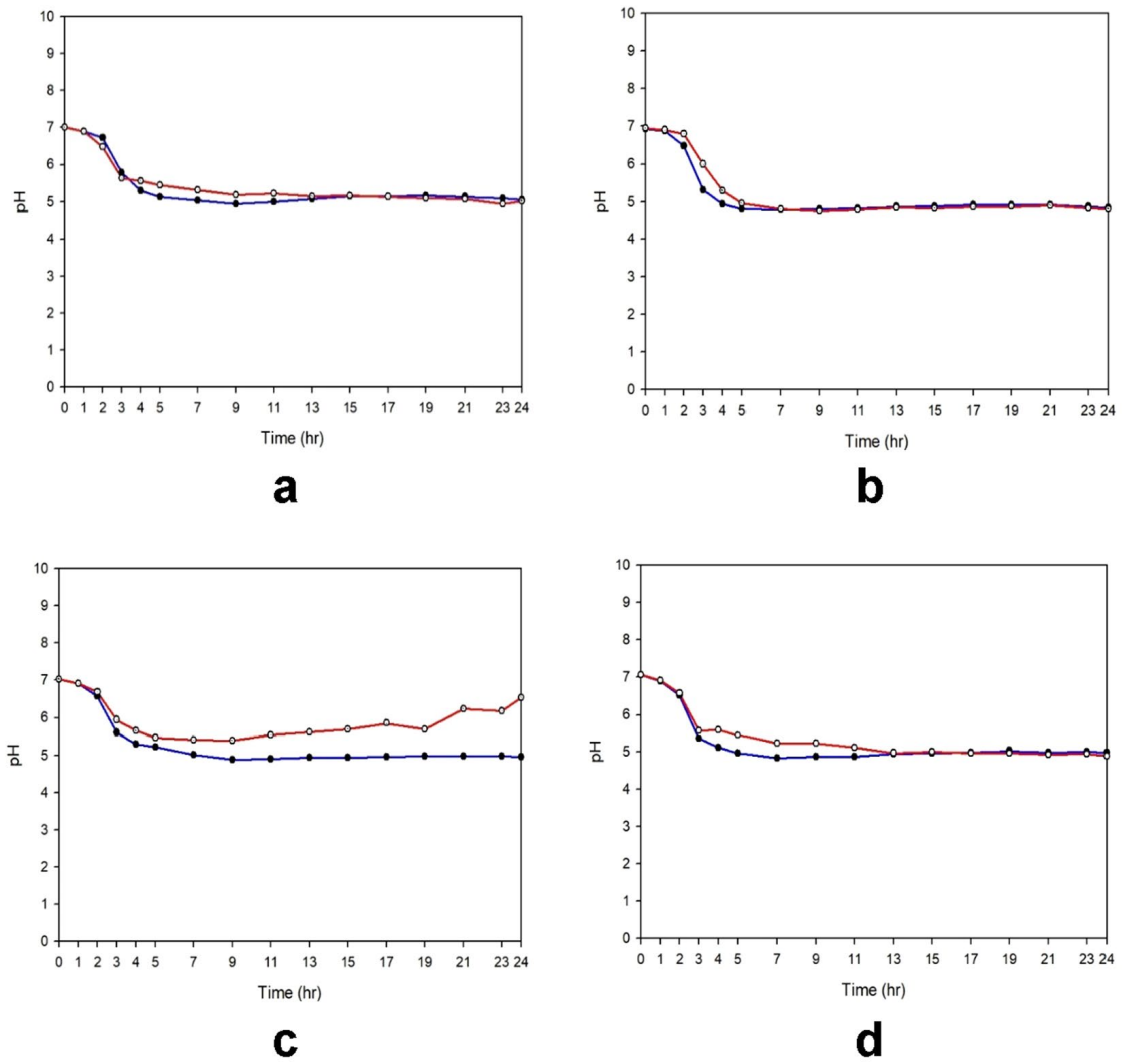
Difference in the rate of decrease in pH in culture media determines the rate of viability loss of *V. cholerae* strains. pH changes in cultures of isogenic strains that contain functional VC1589 or non-functional VC1589 in the presence of 1% glucose were compared. (**a**) N16961 (circle) and DHL001 (filled circle), (**b**) DHL003 (circle) and B33 (filled circle), (**c**) DHL006 (circle) and IB4122 (filled circle), and (**d**) DHL007 (circle) and IB5230 (filled circle).

We have determined that the rate of pH decrease in the presence of glucose differed between these groups. At 1% glucose, the rate of pH decrease in the first group (viability loss within 9 hrs) was faster than in the second group (viability loss in the late stationary phase) (Figs. 4 and S4). This result indicates that the acidity still increases even in strains that can perform neutral fermentation and that the rate for attaining a certain critical pH influences the rate of loss of viability in *V. cholerae* strains.

### Determination of critical pH for loss of viability in *V. cholerae* strains

**Figure 5:**
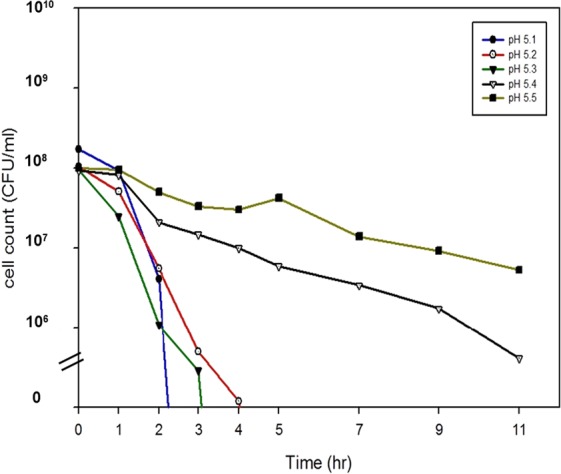
Determination of critical pH for loss of viability of *V. cholerae*. Approximately 10^8^ cells of a Wave 1 strain, N16961, and a Wave 3 strain, IB5230, grown in LB were harvested and resuspended in 0.1 M citrate buffer solution, the pH of which was adjusted to 5.1 (filled circle), 5.2 (circle), 5.3 (inverted filled triangle), 5.4 (inverted triangle), and 5.5 (filled square). Viable cells were counted on LB plates for up to 24 hours. Data were obtained from three independent experiments and a representative result of IB5230 is shown.

Because the rate of loss of viability in *V. cholerae* strains is linked to that of the pH in the culture, we anticipated that we could determine a critical pH for the viability of *V. cholerae* strains. Approximately 10^8^ cells were resuspended in 0.1 M citrate buffer solutions, the pH of which was adjusted to 5.1–5.5 (N16961and IB5230 were tested as they contained functional and nonfunctional VC1589, respectively). *V. cholerae* strains lost viability within 4 hours when the pH was maintained under pH 5.3 but survived for up to 24 hrs at above pH 5.4 (Fig. 5). This result implies that pH 5.3~5.4 is critical for the loss of viability in *V. cholerae* and that the rate at which the pH declines to this critical level determines the time at which viability begins to decrease in the presence of glucose.

### Loss of viability in the presence of glucose is unique in *V. cholerae*

*V. cholerae* strains are acid-sensitive^18^. To confirm that the loss of viability occurrs only in *V. cholerae* by acidification of culture media, 2 other bacterial species—*Escherichia coli* DH5α and *Salmonella* Typhi CT18—were subjected to the same experimental settings^19,20^. Similar pH changes in the culture media were observed, except that the initial decrease in pH in the first 3 hours was not seen in *E*. *coli* or *S*. Typhi (Supplementary Fig. S5). The pH eventually reached pH 4.5 for both bacteria in the presence of glucose, but they maintained their viability for up to 24 hrs. Thus, acid-producing metabolism under glucose-supplemented culture conditions is common in bacterial species, but only *V. cholerae* strains lose viability from acidification.

## Discussion

Among *V. cholerae* O1 serogroup strains, classical biotype strains lose viability through organic acid-producing fermentation under glucose-rich conditions^5^. El Tor biotype strains—Wave 1 strains, at least—have an alternative fermentation pathway that generates 2,3-butanediol, such that they can maintain a neutral pH and survive in the presence of glucose^5^. In this report, we found that more complicated glucose metabolism choices exist in *V. cholerae* El Tor biotype strains, based on the availability of glucose in the media.

The type of glucose metabolism and the outcomes vary, depending on the glucose availability and growth phase of the bacteria. Wave 1 El Tor biotype strains lose viability in the late stationary phase (17 hrs) when grown in glucose-abundant media, perhaps due to the organic acids that they produce. Moreover, current global atypical El Tor strains (Wave 2 and 3 strains) lack neutral fermentation and behave as classical biotype strains in glucose-supplemented media.

The phenotypic alteration in Wave 2 and 3 atypical El Tor strains is attributed to the loss of function in VC1589, which encodes α-acetolactate decarboxylase, a key enzyme in neutral fermentation. The disruption of neutral fermentation by the loss of function in VC1589 was confirmed by phenotype restoration, wherein nonfunctional VC1589 of atypical El Tor strains was replaced with functional VC1589 of Wave 1 strains, and vice versa.

The classical biotype strains and Wave 2 and 3 atypical El Tor strains that have nonfunctional VC1589 continue acidic fermentation when glucose is present. The declines in pH and viability occur quickly in these strains when glucose is supplemented.

Wave 1 prototype El Tor strains and some derivatives of Wave 2 and Wave 3 strains that contain functional VC1589 (in this study, strains MG116025 and IB5230, respectively) survive for up to 24 hours with limited glucose (0.5%) in the media. The pH of the culture media falls slightly for the first 3 hours and then recovers to 8, as in regular LB media. However, in a high-glucose environment (1.0% and 1.5%), the pH of the media decreases to 5, and the bacteria lose viability in the late stationary phase (17 hours). Perhaps the bacteria resume acidic fermentation after a certain degree of neutral fermentation, consequently allowing the acidic byproducts to overwhelm the neutral fermentation products.

In this study, a single-base deletion in VC1589 in 5 Wave 2 and 3 El Tor strains was identified by Sanger sequencing. We also verified, based on this deletion in whole-genome sequencing data, that the preference for acidic fermentation is a characteristic of most Wave 2 and 3 El Tor strains^11,21,22^. From the mid-1990s, Wave 2 and 3 El Tor biotype strains have supplanted Wave 1 El Tor strains globally, suggesting that the acidic fermentation phenotype in classical biotype strains and Wave 2 and 3 El Tor strains is not more detrimental to *V. cholerae* than prototype El Tor biotype strains^3^.

One of the hallmarks during the population changes in *V. cholerae* was the CTB subunit, from classical type CTB (*ctxB1*) in classical biotype strains to El Tor type CTB (*ctxB3*) in prototype El Tor strains and then to *ctxB1* or *ctxB7* in atypical El Tor strains^3,23^. However, this alteration in carbohydrate metabolism should also be considered an important factor in population changes.

To determine the exact choice of fermentation pathway and the composition of fermentation byproducts, the metabolites in culture media at critical points should be analyzed, such as when the pH drops to 6 during the first 3 hrs, when the pH recovers to 8 in LB media, and when it decreases to 5 and bacterial viability is lost in the presence of glucose, providing insight into metabolism shifts in *V. cholerae*.

Wave 2 and 3 El Tor strains contain nonfunctional VC1589, and thus, the drop in pH and loss of viability occur more rapidly in the presence of glucose than in Wave 1 El Tor strains. Based on this property, we should reconsider oral rehydration treatment for cholera patients^2^. By adjusting the amount of glucose in oral rehydration solutions, it might be possible to facilitate acidic fermentation and the loss of viability of Wave 2 and 3 El Tor strains in the intestine. The inhibition of intestinal colonization of El Tor biotype strains when co-inoculated with acid-producing *E*. *coli* in a zebrafish model in the presence of glucose demonstrated that the creation of an acidic environment may aid in the treatment of *V*. *cholerae*^24^.

Conversely, acid-producing strains, classical biotype strains, and Wave 2 and 3 strains are more virulent than prototype El Tor biotype strains that produce neutral byproducts^25,26^. The acid-producing features might be important in the virulence and pathogenicity of *V. cholerae*.

Based on our results, *V. cholerae* strains eventually die from acidic byproducts in glucose-supplemented media, regardless of their ability to undergo neutral fermentation. Perhaps *V*. *cholerae* strains lose viability by producing organic acids through glucose in human hosts. The water loss that is induced in the human intestine by the activity of CT might mitigate the acidification of the intestinal environment by acidic fermentation. *V. cholerae* might have utilized CT for greater survival in human hosts.

## Materials and Methods

### Bacterial strains

A *V. cholerae* classical biotype strain, O395; 2 Wave 1 El Tor biotype strains, N16961 and T19479; 3 Wave 2 atypical El Tor strains, B33, MJ1236, and MG116025; and 2 Wave 3 strains, IB4122 and IB5230, were analyzed^15,27^. Each *V. cholerae* strain and its derivatives are listed in Table 1. *E. coli* DH5α and *S*. Typhi CT18 were also used to compare growth in media that was supplemented with glucose^20,28^.

**Table 1:** *V. cholerae* strains used in this study.

Strains	Biotype	VC1589 genotype	Genome sequence information
O395	Classical biotype		CP000626/CP000627
N16961 derivatives	Wave 1 El Tor strain		AE003852/AE003853
N16961		VC1589-T7	
DHL001		VC1589-T6	
T19479 derivatives	Wave 1 El Tor strain		ERS013250
T19479		VC1589-T7	
DHL002		VC1589-T6	
B33 derivatives	Wave 2 El Tor strain		ACHZ00000000
B33		VC1589-T6	
DHL003		VC1589-T7	
MJ1236 derivatives	Wave 2 El Tor strain		CP001485/CP001486
MJ1236		VC1589-T6	
DHL004		VC1589-T7	
MG116025 derivatives	Wave 2 El Tor strain		ERS013135
MG116025		VC1589-T6	
DHL005		VC1589-T7	
IB4122 derivatives	Wave 3 El Tor strain		ERS013264
IB4122		VC1589-T6	
DHL006		VC1589-T7	
IB5230 derivatives	Wave 3 El Tor strain		AELH00000000.1
IB5230		VC1589-T6	
DHL007		VC1589-T7	

VC1589-T7: functional VC1589, VC1589-T6 non-functional VC1589.

*toxT*-Y: amino acid 139 of *toxT* is Tyr, *toxT*-F: amino acid 139 of *toxT* is Phe.

### Sequencing of VC1589 and VC1590

A 2153-bp DNA fragment, encompassing VC1588 and VC1589 from Wave 1 El Tor biotype strains, and a 2152-bp fragment, harboring VC1588 and VC1589 from Wave 2 and 3 strains, were amplified by primer set 1588 F: CAA CGC CTG ATA CTT GGA AG and 1589 R: CGA TAG GTG GAT GGG CAC TTC C. A 2210-bp DNA fragment that contained VC1590 from Wave 1, 2, and 3 El Tor strains was amplified using primer set 1590 F: GGA CGG GTG GCG GAC ACA TTC and 1590 R: CTT GCA CCT GTT CGG GAT CC. DNA sequences of these fragments were analyzed by dideoxyribonucleotide chain termination method.

### VC1589 allele exchange

We used primer set VC1589 XbaI F: CGG TCT AGA CGG CGG GCG TCA ACT CAA CG and VC1589 SacI R: CGG GAG CTC TTG GGC GAT AAG TTG TGC TC, to generate a 1261-bp fragment that contained functional VC1589 from the Wave 1 El Tor biotype strain N16961 and a 1260-bp fragment that encompassed nonfunctional VC1589 from the Wave 2 atypical El Tor strain B33, which were subcloned in suicidal plasmid pCVD442 to construct pCV442-VC1589-T7 and pCVD442-VC1589-T6, respectively. pCVD442-VC1589-T7 was conjugally transferred to Wave 2 and 3 atypical El Tor strains to replace their nonfunctional VC1589 with functional VC1589. Similarly, functional VC1589 in Wave 1 El Tor biotype strains was replaced by allele exchange method with pCVD442-VC1589-T6. Allele replacement with VC1589 was confirmed in each strain by sequencing.

### Bacterial viability and pH changes in bacterial culture

Overnight cultures of each bacterial strain were inoculated into fresh media at an optical density of 0.01 (approximately 10^7^ cells/ml). Glucose was added to a final concentration of 0.5%, 1.0%, and 1.5% using sterile 50% glucose stock solution. Viable cells were counted on LB plates. Viable cell counts and pH were measured every hour for the first 10 hours and every 2 hours for the next 14 hours. A benchtop pH meter (Thermo Scientific, Waltham, MA) was used to measure the pH of media.

## Supplementary information

Supplementary Figures s1-s5.
