# Supplementary material for: Alterations in glucose metabolism in Vibrio cholerae serogroup O1 El Tor biotype strains
Source: Sci Rep. 2020 Jan 15;10:308. doi: 10.1038/s41598-019-57093-4 (PMC6962216; doi:10.1038/s41598-019-57093-4)

Alterations in glucose metabolism in *Vibrio cholerae* serogroup O1 El Tor biotype strains

Donghyun Lee[1,2¶], Eun Jin Kim[1,2¶], Yeongjun Baek[1,2], Jiwon Lee[1,2], Youngbae Yoon[1,2], G. B. Nair[3], Sang Sun Yoon[4,5,6*], and Dong Wook Kim[1,2*]

[1]Department of Pharmacy, College of Pharmacy, Hanyang University, Ansan 15588, Republic of Korea

[2]Institute of Pharmacological Research, Hanyang University, Ansan 15588, Republic of Korea

[3]Microbiome Laboratory, Rajiv Gandhi Centre for Biotechnology, Kerala 695014, India

[4]Department of Microbiology and Immunology, Yonsei University College of Medicine, Seoul 03722, Korea

[5]Brain Korea 21 PLUS Project for Medical Sciences, Yonsei University College of Medicine, Seoul 03722, Korea

[6]Institute for Immunology and Immunological Diseases, Yonsei University College of Medicine, Seoul 03722, Korea

*Correspondence:
Sang Sun Yoon
sangsun_yoon@yuhs.ac

Dong Wook Kim
dongwook@hanyang.ac.kr

# Supplementary Fig. S1.

**Supplementary Fig. S1. Growth curve (left) and pH changes in cultures (right) of *V. cholerae* strains in glucose-supplemented media.** (a) Wave 1 prototype El Tor strain T19479, (b) Wave 2 atypical El Tor strain MJ1236, (c) Wave 2 atypical El Tor strain MG116025, and (d) Wave 3 atypical El Tor strain IB4122. Glucose was added to LB media at 0% (filled circle), 0.5% (circle), 1.0% (inverted filled triangle), and 1.5% (inverted triangle). Values of mean ± standard deviation (viable cell count) and a representative result (pH) are shown.

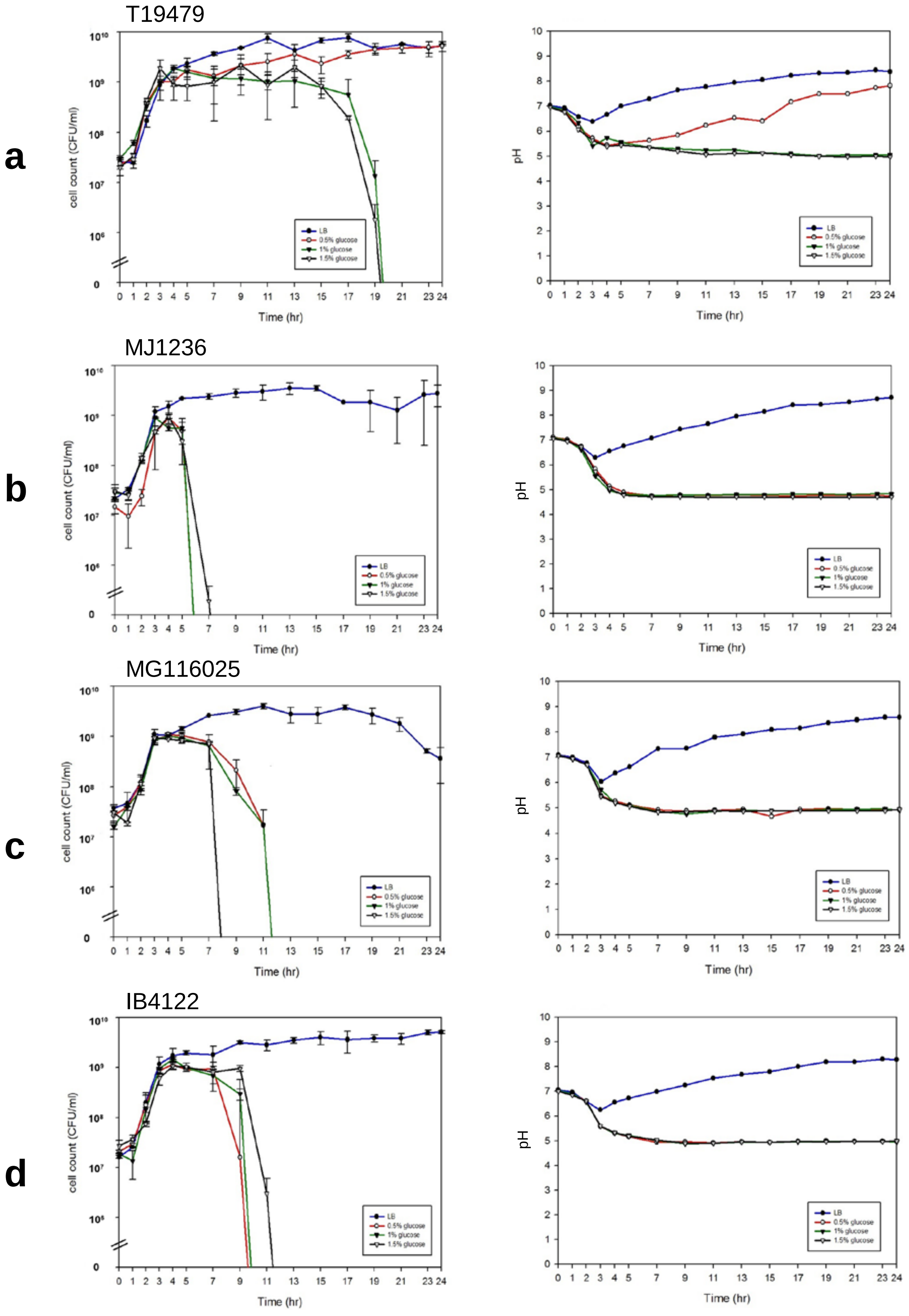

# Supplementary Fig. S2.

**Supplementary Fig. S2. Alterations in glucose metabolism in El Tor biotype strains by a single-base deletion in VC1589: Wave 1 and 2 strains.** Growth curve (left) and pH changes in cultures (right) of El Tor biotype strains in glucose-supplemented media. (a) Derivative of Wave 1 El Tor strain T19479 containing nonfunctional VC1589 (strain DHL002) and (b) derivative of Wave 2 atypical El Tor strain MJ1236 that contained functional VC1589 (strain DHL004). Values of mean ± standard deviation (viable cell count) and a representative result (pH) are shown.

DHL002

a

b

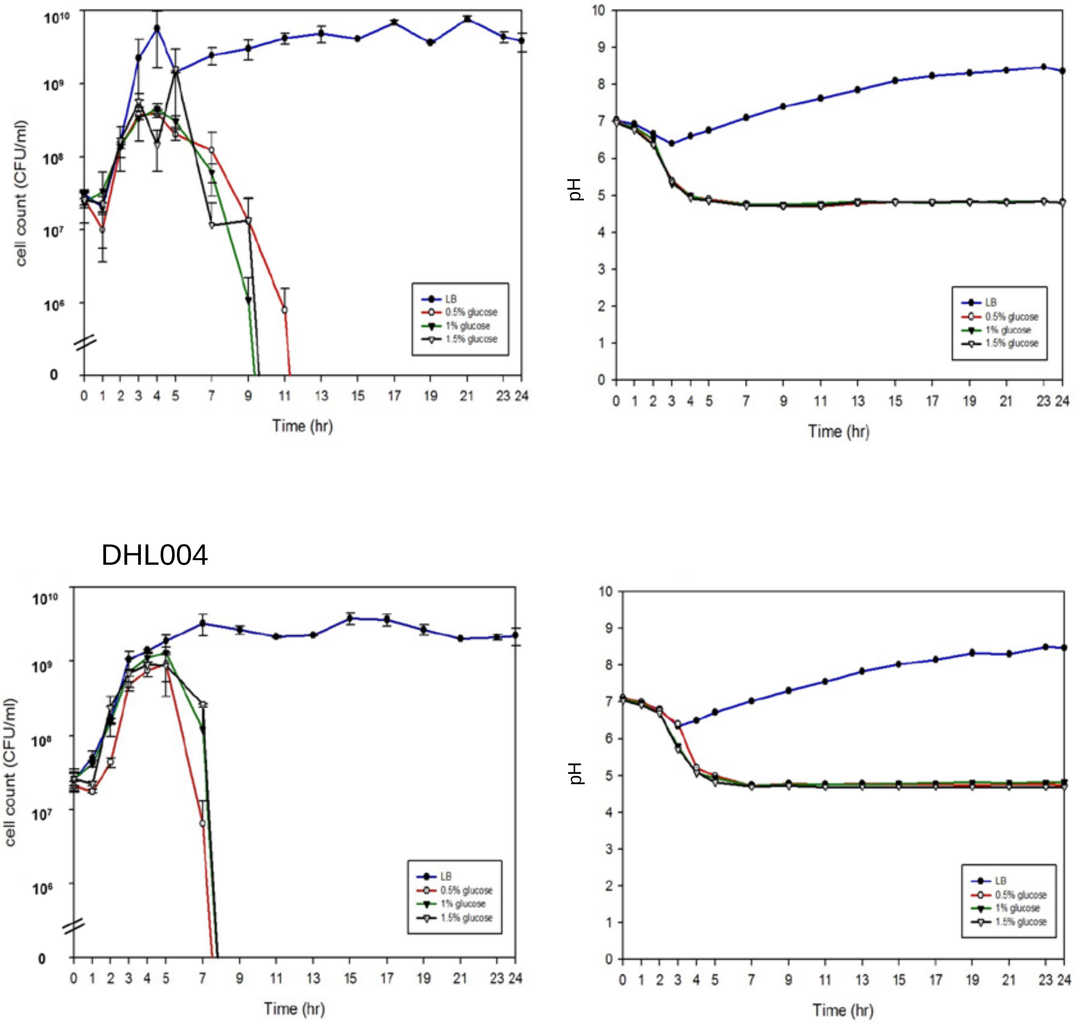

# Supplementary Fig. S3.

**Supplementary Fig. S3. Comparison of viability of the strains that contain nonfunctional VC1589 (left) and strains that harbor functional VC1589 (right) at different glucose concentrations.** Viable cell counts in LB (a), LB supplemented with 0.5% glucose (b), 1.0% glucose (c), and 1.5% glucose (d). Bacterial viability results of Fig. 1 – 3 and Supplementary Fig. S1 – S2 are combined.

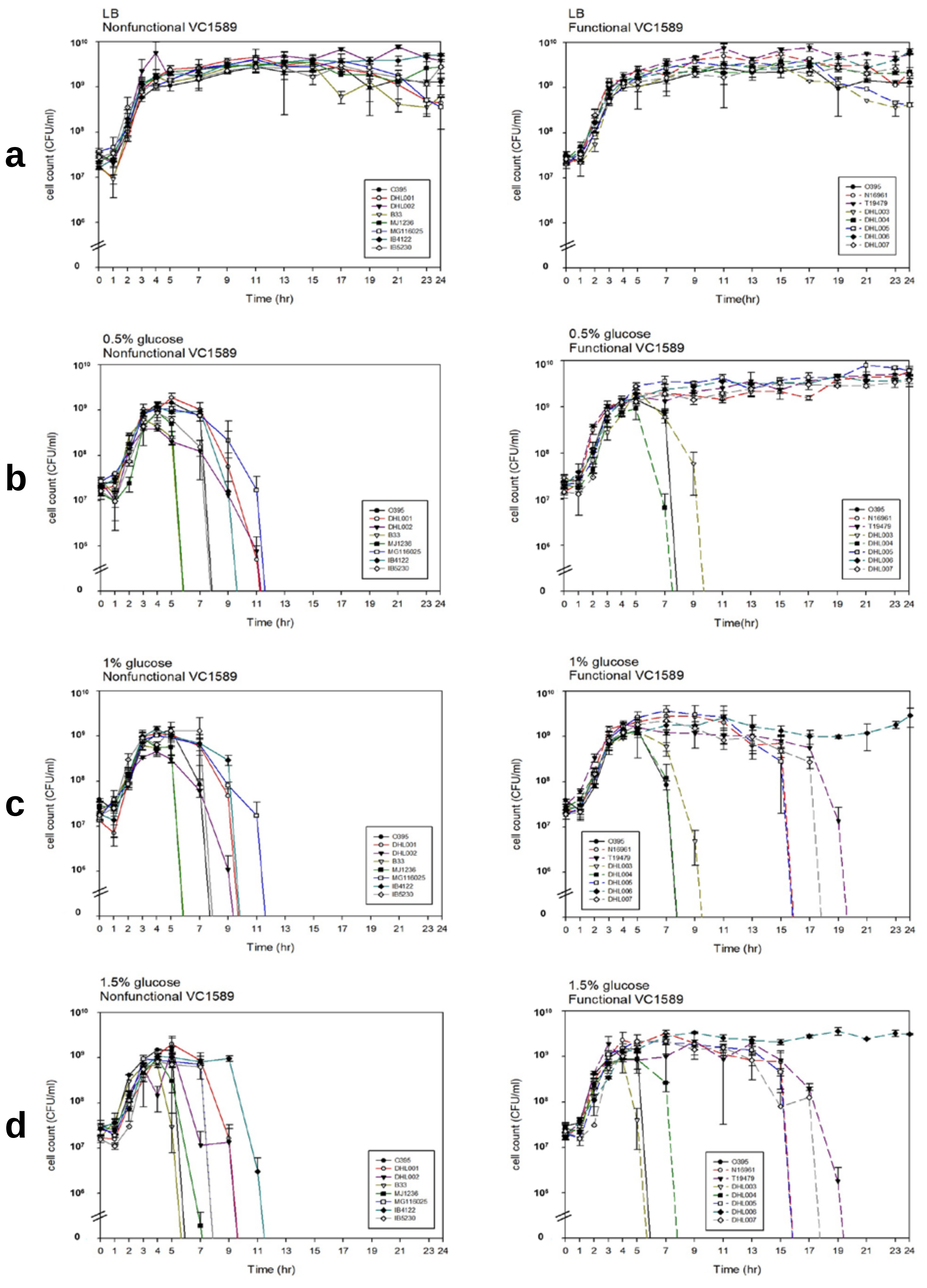

# Supplementary Fig. S4.

**Supplementary Fig. S4. Difference in the rate of decrease in pH in culture media determines the rate of loss of viability of *V. cholerae* strains.** The pH changes in cultures supplemented with 1% glucose were compared for (a) DHL002 (filled circle) and T19479 (circle), (b) MJ1236 (filled circle) and DHL004 (circle), and (c) MG116025 (filled circle) and DHL005 (circle).

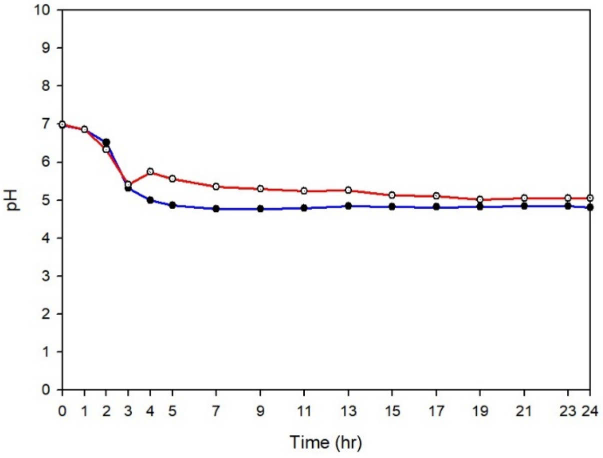


**a**

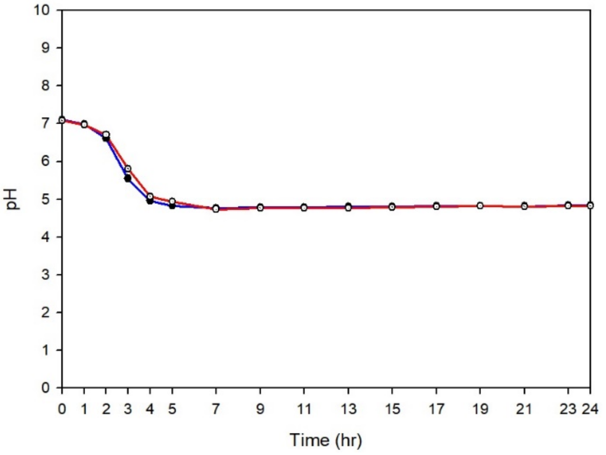


**b**

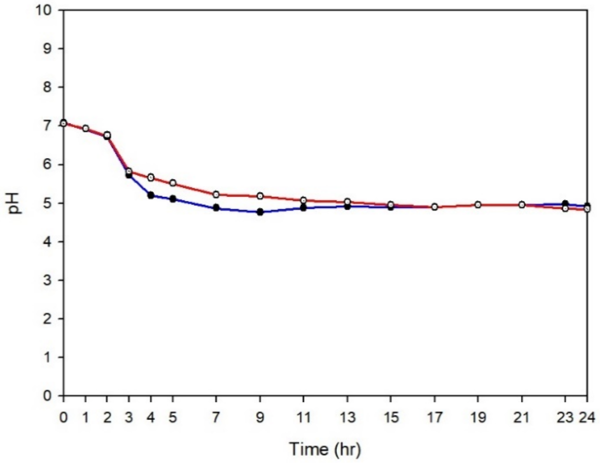


**c**

# Supplementary Fig. S5.

**Supplementary Fig. S5. Growth curve (left) and pH changes in cultures (right) of *E. coli* DH5α (a) and *S*. Typhi (b) in glucose-supplemented media.** Glucose was added to LB media at 0% (filled circle), 0.5% (circle), 1.0% (inverted filled triangle), and 1.5% (inverted triangle). Values of mean ± standard deviation (viable cell count) and a representative result (pH) are shown.

*E. coli*

a

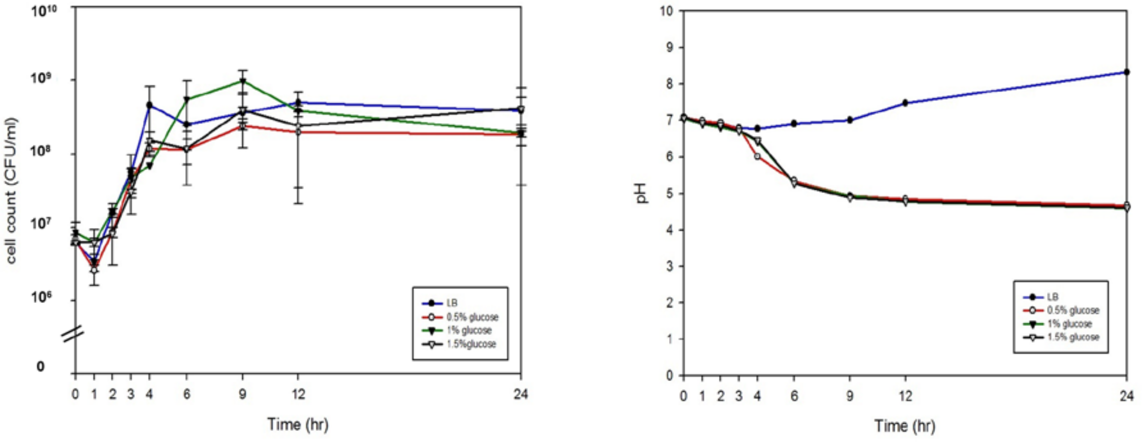


*S.* Typhi

b

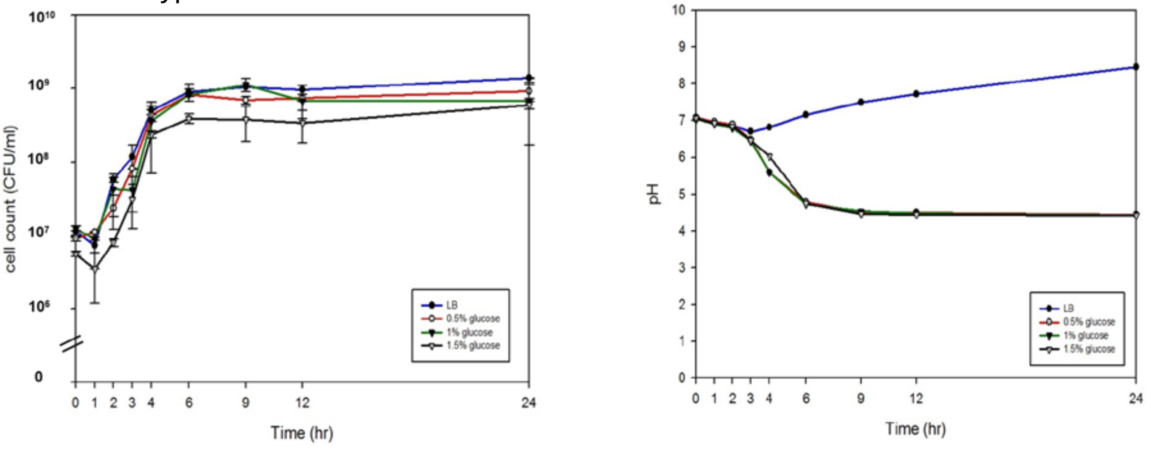

Supplement: Supplementary file 1 — Supplementary Figures s1-s5. [file 41598_2019_57093_MOESM1_ESM.pdf]
